# What have we learned so far from the sex/gender issue in heart failure? An overview of current evidence

**DOI:** 10.1007/s11739-022-03019-4

**Published:** 2022-06-30

**Authors:** Michele Arcopinto, Valeria Valente, Federica Giardino, Alberto Maria Marra, Antonio Cittadini

**Affiliations:** 1 Department of Translational Medical Sciences, “Federico II” University Hospital and School of Medicine, Via Sergio Pansini, 5, 80131 Naples, Italy; 2 Center for Pulmonary Hypertension, Thoraxklinik at Heidelberg University Hospital, Heidelberg, Germany; 3 Interdepartmental Centre for Biomaterials (CRIBB), “Federico II” University, Naples, Italy

**Keywords:** Heart failure, Sex, Gender, Males, Females

## Abstract

There are important differences in epidemiology, pathophysiology, HF patterns, prognosis, and treatment. Women have a higher incidence of HFpEF due to sex-specific factors (such as anthropometry, role of estrogens, pregnancy-related cardiomyopathies), increased incidence of comorbidities, and gender-specific conditions. Men instead present a predisposition to the development of HFrEF due to a higher incidence of coronary artery disease and myocardial infarction. However, there are still gaps in the management of women with HF. The poor inclusion of women in clinical trials may have contributed to a lesser understanding of disease behavior than in men. In addition, a full understanding of gender-specific factors that are studied in small populations is lacking in the literature, and only in recent years, studies have increased their focus on this issue. Understanding how society, family, and environment affect the prognosis of HF patients may help clinicians provide more appropriate levels of care. In this overview, we aimed at summarizing all the key available evidence regarding sex/gender differences in heart failure.

## Introduction

Chronic HF represents the endpoint of virtually all adult chronic cardiovascular diseases and is often secondary to pre-existing conditions, among which ischemic heart disease, systemic arterial hypertension, diabetes mellitus, and atrial fibrillation play a major role. These clinical conditions and other risk factors show a different prevalence according to sex and may have different weights concerning the development of a specific phenotype of HF. Similarly, the clinical presentation and the pathophysiologic aspects of HF, the response to pharmacologic and electrophysiological therapy, as well as the prognosis of HF may vary in different sexes [1].

The overall lifetime risk of HF is quite comparable between the sexes; estimated at 21% for men and 20% for women at the age of 40 years in the Framingham Heart Study (FHS) [2], and 33% for men and 29% for women at age of 55 years in the Rotterdam Study [3]. However, the study of HF in women has been underestimated for many years, mainly because of a believed better prognosis/delayed onset in the life of such patients compared with men [4].

Furthermore, there is an underrepresentation of women in Randomized Clinical Trials (RCT) in HF, although improving over time [5], and a paucity of studies specifically targeting women with HF.

Alongside specifically clustered sex-related differences, gender is a more complex concept encompassing mainly four domains: (1) gender roles (the behavioral norms applied to males and females), (2) gender identity (how people describe themselves as female or male), (3) gender relations (how people interact with or are treated according to the ascribed gender), (4) institutionalized gender (distribution of power in the social life in terms of political, education and social institution) [6].

This overview aims to summarize the most recent evidence regarding differential clinical presentation, management of risk profile, and therapy in HF to increase awareness of differential approaches according to patient sex/gender to maximize the outcome and optimize the use of health resources.

## Clinical risk for heart failure development

The risk for incident HF includes some well-known, not modifiable factors, such as age, family history of cardiovascular disease, and ethnicity. Although these risk factors are nonmodifiable, they may have a different weight for male and female patients. Regarding “traditional” modifiable risk factors like systemic arterial hypertension, type 2 diabetes mellitus (T2DM), obesity, smoking habit, and renal impairment, these may have different prevalence in males and females and, even when assuming similar prevalence for some of them, they still may have a different weight in the overall risk of developing HF.

Virtually, all the mentioned risk factors contribute to the development of ischemic heart disease which, in turn, is the most common condition underlying HF in the general population.

In a subanalysis of the Prevention of REnal and Vascular ENdstage Disease (PREVEND) study [7], which primarily addressed the impact of albuminuria on future cardiovascular and renal disease, 8592 middle-aged subjects with a mean age of 49.2 (50.1% women) were followed-up for a median of 12.5 years and the incident HF and the cardiovascular risk profile was specifically measured and compared between men and women. Among the 374 subjects who developed new-onset HF, 241 (64.4%) were men and 133 (35.6%) were women. Men developed heart failure earlier than women (7.0 vs. 8.6 years) and were slightly younger at the time of the diagnosis (71.3 vs 72.7 years). As detailed later, the multivariable competing risks analyses showed that women had a lower risk for HF with reduced ejection fraction, HFrEF (sub hazards ratio = 0.47) but a higher risk for HF with preserved ejection fraction, HFpEF (sub hazards ratio = 2.16) than men. In this analysis, among all risk factors, only atrial fibrillation had a sex-specific predictive value and increased risk specifically for women.

Other studies specifically explored the weight of specific risk factors for incident HF in women and men. In a one million person-year, follow-up study performed between 2000 and 2005 on an insured US population of > 350,000 HF-free subjects, a separate prediction model for newly diagnosed HF was developed for each sex [8]. In this cohort, a total of 4001 incident HF cases were recorded with an incidence rate of 3.68 cases per 1000 person-years of follow-up in women and 4.24 in men with an incidence rate of HF higher in men compared with women in any given observation year. As expected, the incidence rate of HF increased markedly with increasing age with men having a higher rate of incident HF in all age groups < 75 years. However, for the ≥ 75-year age group, women had a similar or even slightly higher HF incidence rate than men. As expected, in patients with incident HF, there was a high prevalence of risk factors in both sexes. Among the five risk factors for which the authors examined the prevalence in the population with incident HF (hypertension, diabetes mellitus, coronary artery disease, atrial fibrillation, and valvular heart disease), they found that: (1) 80.4% of women and 76.3% of men had hypertension; (2) 30.5% of women and 31.7% of men had diabetes; (3) nearly 18% of women and 27% of men had coronary artery disease; (4) 8% of women and 10% of men had atrial fibrillation, and (5) 9% of both women and men had valvular heart disease. More than 80% of women and men with incident HF had at least 1 diagnosed HF risk factor, and 14% of women and 19% of men with incident HF had 3 or more antecedent HF risk factors. Each of the five risk factors conferred a substantial risk for incident HF in both women and men in unadjusted models. The hazard ratios associated with the individual factor were similar for age (10-years increase), hypertension, coronary artery disease, diabetes, and valvular disease, whereas it was higher in women regarding atrial 3 fibrillation (HR 16.3 vs 11.3).

The influence of sex on the risk of new-onset HF in patients with known or suspected coronary artery disease (CAD) was examined in 5899 HF-free consecutive patients (38.8% women) with known or suspected CAD undergoing vasodilator stress cardiac magnetic resonance [9]. After a median follow-up of 4.5 years, 5.3% of this population developed new-onset HF. Unadjusted new-onset HF rates were higher in women than in men (1.25 vs. 0.83 per 100 person-years) and after multivariate adjustment, women showed an increased risk of new-onset HF (hazard ratio 1.58).

Sex differences have been described in T2DM cardiovascular outcome trials. A meta-analysis involving > 40,000 participants with T2DM from five studies [10] showed a significant underrepresentation of women in these trials (28.5–35.8%), and overall, a greater prevalence of HF with a 1.3 relative risk compared to men.

The finding of systemic arterial hypertension as a leading risk factor for HF is not new as well as it is the greater incidence of HF in hypertensive women compared to hypertensive men. In this regard, a total of 5143 subjects aged 40–89 years and free of HF from the Original Framingham Heart Study were followed-up for a mean period of 14.1 years. In 91% of the diagnosed HF cases, hypertension antedated its development [11]. Adjusting for age and other HF risk factors the hazard for developing heart failure in hypertensive compared with normotensive subjects was about twofold in men and three-fold in women leading to an attributable risk for HF accounting for 39% of cases in men and 59% in women. Taking all together, atrial fibrillation showed in population-based studies to be the only sex-specific risk factor for HF development. To date, no data are available regarding gender-related risk factors, and more research is warranted on this issue.

## The pattern of HF according to sex

According to ESC guidelines, the most common and accepted classification of HF is based on the left ventricular ejection fraction of the left ventricle (LVEF). Three patterns have been recognized, with some specificity in terms of pathophysiology and therapeutic management: heart failure with preserved ejection fraction (HFpEF, LVEF ≥ 50%), heart failure with reduced ejection fraction (HFrEF, LVEF ≤ 40%), and heart failure with mid-range ejection fraction (HFmrEF, LVEF 41–49%) [12].

### HFpEF

The prevalence of HFpEF is about double in women than in men and it is higher at any given age [13, 14]. There are several pathophysiological hypotheses to explain this asymmetric incidence including the difference in anthropometry, a different LV architecture, and EF in healthy males compared with a females, the role of estrogens, differential gene expression, different tendency to maintain a systemic inflammation status, and the pattern of HF comorbidities. All these factors may play together and have a greater influence on coronary microvascular/endothelial inflammation.

In the Dallas Heart Study [15], a CMR study in HF-free individuals of both sexes aged 30–65 years, was found that the median and 25th–75th range in LVEF was higher in women than in men and that LV volumes indexed to body surface area were smaller in women than in men. According to the study, low LVEF (values below the 2.5th percentile of a healthy subset) was defined as below 61% in women and below 55% in men. An intricate hypothesis is that the greater female predisposition to develop HFpEF is due to their commonly reduced ventricular size and stroke volume, although maintain a similar cardiac output compared to men. Furthermore, systolic and diastolic elastance (stiffness) of the LV walls seems to be increased in men, with a tendency to increase with age. Last, but not least, animal studies showed also that the female heart is equipped with a larger pool of functionally competent human cardiac stem cells, younger myocytes, and a faster myocyte turnover than the male myocardium may influence the tendency toward a specific form of ventricular hypertrophy [16].

Therefore, the anatomical variance of LV geometry and function and their respective remodeling might be the pathophysiological bedrock to a different tendency to develop one HF phenotype rather than another one. As for several diseases with a remarkable difference in prevalence by sex, the possible role of estrogens in the development of HFpEF, especially in postmenopausal women, has been extensively explored. The hypothesis is well supported by the fact that estrogens play a wide array of vascular actions including the balance between vasodilation and vasoconstriction, promote angiogenesis, and exert a protective role against fibrosis and oxidative stress. Therefore, recent evidence has hypothesized that the decline in estrogen levels after menopause contributes to myocardial microvascular dysfunction and makes postmenopausal women more vulnerable to microvascular endothelial damage [17]*.*

Although no significant progress has been made with ‘anti-inflammatory’ therapies in HF patients, systemic inflammation is believed to be a contributor to the development of HF [18]*.* In this regard, women are characterized by a greater expression of pro-inflammatory genes, high levels of cytokines, and greater involvement of immunity mediated by T cells [19]. The higher prevalence of autoimmune diseases in women is associated with a greater tendency to low-grade inflammation and in turn endothelial dysfunction—a relevant concept to the development of HFpEF—microvascular impairment and higher incidence of diastolic dysfunction [20].

Obesity is a stronger risk factor for the development of HFpEF than HFrEF, as its risk rises by 34% for every standard deviation increase in body mass index. However, this association is stronger in women than in men [21]*.* Obesity is a risk factor with a higher prevalence in women and obese women are more likely to develop HFrEF than obese men [22]*.*

### HFrEF

The higher risk of HFrEF in men than women has been attributed to their higher tendency to coronary artery disease and myocardial infarction, a well-known antecedent. As outlined before, this has been contrasted with microvascular dysfunction of the coronary circulation that may play a major role in HFpEF. On the other hand, women are more likely than men to develop HF after myocardial infarction [23].

In a prospective observational study involving nearly 30,000 participants followed for incident HF over a median follow-up of 12 years, men had an almost two-fold higher risk than women for HFrEF [24]. In keeping with this observation, in the Swedish Registry “SwedeHF”, women constituted 55% of patients with HFpEF and 29% of patients with HFrEF [25].

In the HFrEF population, special sex-specific predisposing conditions must be considered. Among these, peripartum cardiomyopathy, chemotherapy-induced cardiomyopathy, and Tako-Tsubo cardiomyopathy are non-ischemic etiologies for HFrEF with high specificity for women.

Peripartum cardiomyopathy is exclusive to young women, especially in the last months of pregnancy and the months following childbirth. The risk is increased by multiparity, and multiple gestations and has a strong association with preeclampsia and hypertension in pregnancy. The incidence of this condition ranges from 1 in 1000 to 4 in 1000 live births and marked variability in the incidence by geographical area may reflect a different race susceptibility (as well as different normative criteria) [26]. Almost 13% of women with pregnancy-induced HFrEF do not recover EF in the subsequent 6 months [27].

Post-chemotherapy dilated cardiomyopathy is mainly represented in women for the frequent use of anthracyclines in the treatment of breast cancer, a known cardio-toxic drug. It has been shown that doxorubicin decreases LVEF by about 10–15% at a standard dose of 240 mg/sqm and has a linear relation with cumulative dose. Therefore, data from the literature suggest there is a predilection for cardiotoxicity in women using anthracyclines over men as determined by differences in pharmacokinetics [28].

Tako-Tsubo syndrome, also known as the "broken heart" syndrome is an acute transient condition with a 9:1 ratio for incidence in favor of women. Although the etiology is unclear, attention has been paid to the role of catecholamines overstimulation causing direct toxicity, and microvascular vasoconstriction associated or not with coronary artery spasm [29].

### HFmrEF

Based on the results of recently published clinical studies, it is estimated that the percentage of HFmrEF patients is about one-third of the whole HF population [30]. Evidence from the literature has shown a clinical behavior similar to HFpEF, as it is associated with ischemic coronary disease in two-thirds of the population [31].

In the APOLLON study addressing a total of 246 HFmrEF patients, 57.7% were male. Compared to women, younger men had lower BMI, were more frequently smokers, and were more frequently affected by coronary heart disease. Among women, there was a higher prevalence of atrial fibrillation and arterial hypertension [32]. The higher percentage of male patients in the HFmrEF population is confirmed by a larger study by Bhambhani et al., which enrolled 28,829 HF-free individuals of both sexes and followed-up for 12-year, identifying a percentage of women developing HFmrEF equal to 48% [33].

However, it is worth noting that several authors have regarded the HFmrEF population as a heterogeneous group of patients with HFrEF or HFpEF with a change in LVEF over time. Some observations suggest that the group of patients with improved LVEF should constitute a distinctive category of HF (HF with improved EF, HFiEF) [34].

## Treatment differences in the real world

Pharmacological treatment of patients affected by HF consists in mitigating the symptoms of pulmonary congestion, such as dyspnea, cough, and peripheral edema, and improving prognosis and survival chances. To raise survival chances, the ESC guidelines suggest the use of angiotensin-converting enzyme inhibitors (ACE-I)/angiotensin receptor blockers (ARBs), aldosterone antagonists, or an association of angiotensin receptor and neprilysin inhibitors (ARNI), to oppose cardiac remodeling and the combination of cardiomyocytes fibrosis and apoptosis. The sodium-glucose co-transporter 2 (SGLT2) inhibitors dapagliflozin and empagliflozin, in association with the aforementioned therapy, have recently proven to reduce cardiovascular disease death [12].

Evidence demonstrates significant sex disparities in pharmacokinetics and pharmacodynamics in patients affected by HF. It has been reported a 2.5 times higher concentration of ACE-inhibitors, angiotensin receptor blockers, and beta-blockers in women when compared to men, despite similar dose administration [35, 36]. This can be explained considering the lower renal and hepatic metabolism in women [37, 38]. Furthermore, when given a fixed dose of beta-blockers, women show a greater reduction in heart rate and blood pressure compared to men [39].

In the SwedeHF Registry, there were significant differences in HF-related therapy: females were less likely to receive renin–angiotensin system (RAS) inhibitors but more likely to be treated with diuretics regardless of EF. However, after extensive adjustments, there were no sex-based differences in the use of RAAS inhibitors, whereas females were more likely to receive beta-blockers and digoxin across the whole EF spectrum of HF [40].

In men, the risk reduction of all-cause death or HF hospitalization is directly proportional to their titration to the guideline’s target dose. Women, on the contrary, can reach the peak benefit, characterized by a 30% lower risk of all-cause death and hospitalization, using beta-blockers at the dose of 50–60% of the target dose or using ACE-I/ARBs at the dose of 40–60% of the target dose [41]. For patients belonging to the HFpEF group, the PARAGON-HF trial shows that the use of Sacubitril/Valsartan has more benefits in women than in men [42].

In the Digitalis Investigation Group (DIG) trial, it was shown that a higher risk of death, consequent from the use of digoxin, is associated with women [43], due to a higher concentration of drug in their blood even though in the trial, a higher oral daily dose was given to men.

Along with the pharmacological therapy, patients with HFrEF with severely reduced EF require the aid of an implantable cardioverter-defibrillator (ICD) to prevent sudden cardiac death. Additionally, cardiac resynchronization therapy (CRT) improves prognosis in patients with wider QRS complex at EKG whenever dilated cardiomyopathy occurs. Men are more likely to receive an ICD in comparison with women even after adjusting for age and comorbidity. In a very large analysis of patients of both sex with HF and LVEF < 35%, women were less likely to receive counseling for ICD (about 19% versus 25%) although the rate of patients receiving an ICD placement was about 63% for both men and women [44]. In a meta-analysis of five randomized clinical trials for primary prevention with ICD in HFrEF, there was no survival benefit in women randomized to ICD compared to a − 22% in mortality in men [45]. Moreover, women were less likely to receive an appropriate ICD shock [46]. Most CRT implantations are performed along with an ICD (CRT-D) and women were less likely to receive a CRT-D device than men, although left bundle branch block is more common in females than in males [47]. However, CRT benefits were found to be greater in women than men in terms of improved reverse remodeling, quality of life, cardiovascular hospitalization, and overall survival [48, 49].

## Differential prognosis of HF

The HF prognosis is determined by several factors: etiology, left ventricular function, the timing of diagnosis, optimization of pharmacological therapy and individual response to it, and other comorbidities. According to the Framingham Heart Study, between 1990 and 1999, the age-adjusted 5 years cardiovascular mortality rate amounted to 45% in women and 59% in men [50]. In the following decade, as affirmed by the population-based Olmsted County study for incident HF [1], the age-adjusted all-cause mortality rates were found to be similar among the two genders, but the cardiovascular death rate was higher in men than in women. In the SwedeHF Registry, the mortality/HF hospitalization rates after adjustments for confounders were significantly lower in females regardless of EF (HR: 0.80 in HFrEF, HR: 0.91 in HFmrEF, and HR: 0.93 in HFpEF) [25]. With specific regard to HFpEF, the reduction in terms of risk of death or hospitalization has been addressed by the I-PRESERVE trial—originally designed to study the impact of irbesartan administration in HFpEF patients—which estimated a difference of around 20% in favor of women [51]. Since women are more symptomatic, i.e. greater exercise limitation, they have worse quality of life (QoL) but, at the same time, they showed a better prognosis in terms of all-cause mortality and hospitalization [52].

## Lack in evidence

Traditional risk factors for HF are extensively addressed in the literature but there is little evidence about gender-specific determinants of health [53].

Several previous studies have identified determinants, such as socioeconomic status, race, and ethnicity, social support, culture and language, access to care, marital status, and residential environment, as important predictors of disparities in cardiovascular risk. However, variables have not always been studied comprehensively on an evidence-based approach [54, 55].

Socioeconomic determinants represent a major challenge from both a health and social perspective.

Hung et al. [56], in a population of 633 098 hospitalized HF identified by Taiwan National Health Insurance Research Database and divided by type of income, recognized that low-income patients with HF had nearly a twofold increase in the risk of in-hospital mortality and post-discharge events compared with the high-income group, in part because of lower GDMT use. The low-income population was more likely to be female (51.5% female vs 48.5% male), older, and with more comorbidities in contrast to the high-income population, which was predominantly male (78.4 male vs 21.6 female), younger (58.9 years vs 74.6), and with fewer comorbidities.

Although increased in the low-income population, the rate of rehospitalization was mitigated after the implementation of universal health coverage at the national level.

In the United States, similar results were achieved in 2014 with the Affordable Care Act, which expanded Medicaid eligibility in the United States for millions of low-income adults.

In a cohort of 58,804 US HF hospitalized patients (39.18% female vs 60.82% male) analyzed retrospectively, the expansion of coverage led to increased access to care with the implementation of appropriate therapy utilization and medication adherence. The authors hypothesize that implementation of care according to guidelines allowed a reduction in indication for implantation of indicated cardiac devices (i.e., implantable cardioverter defibrillator, cardiac resynchronization therapy). [57]

However, in the study by Thakkar et al. [58], the use of substantial life-saving therapies was compared in 292,070 patients with HF hospitalizations with Medicaid and private insurance (PI), respectively. Patients covered by Medicaid protection were predominantly male, (56.1% men vs 43.9% women), African Americans (42% African Americans vs 32% Whites), smokers (44.6% vs 35.3.PI), and drug users (15.3% vs 2.7%). Patients privately insured patients turned out to be a greater number of males (57.3% vs 42.7% females), white (57.3% vs 42.7% other races), had a higher incidence of coronary artery disease and coronary bypass surgery (16.5% vs 11.1%).

The authors identified that 36% were less likely to use mechanical support devices (IABP, pLVAD, ECMO), 16% were less likely to use implantable devices such as ICD, CRT, PPM, 37% were less likely to perform coronary angiography, 65% were less likely to perform heart transplantation in patients with Medicaid compared with private insurance.

Recent evidence has instead recognized the level of education as a heavier risk factor for the development of HF than economic income in the working phase of life.

The Copenhagen City Heart Study, in which 8616 men and women prospectively participated with a mean follow-up of 12 years, assessed the impact of education on the incidence of HF analyzed.

Education level and family income were significantly associated with the risk of HF (HR) at 0.55 (0.46–0.68) and 0.81 (0.68–0.96) for education level and high or low income, respectively, after adjusting for age, sex, and time period.

Indeed, household income does not accurately reflect socioeconomic position after retirement, whereas educational level, in both sexes, validly represents socioeconomic position from relatively early in life, representing a measure of socioeconomic status over the life course and an important predictor for hospitalizations for HF [59].

Another indicator that is necessary to consider in the prognosis of HF is family status.

Positive social support is associated with a better quality of life [60], while the absence of such support is associated with a worse prognosis and an increased rate of hospitalization [61].

In a recent study, Chung et al. [62] analyzed 723 HFrEF patients whose marital status was collected.

Widowed patients had a higher risk of HFrEF (*p* = 0.047), were older, more frequently female, and belonged to advanced NYHA class (NYHA III-IV), with more comorbidities and less access to care.

The role of positive family support also appears to mitigate the effects of depression in the HF population.

In a study that evaluated 166 HF patients with and without depressive symptoms, the levels of such were similar between married and unmarried patients (10.9 vs. 12.1, *p* = 0.39). However, married patients had longer event-free survival than unmarried patients (*p* < 0.008).

Patients with symptoms of depression were more frequently male, younger, less educated, less financially secure, and more likely to take antidepressants than those without depressive symptoms and with advanced NYHA class (NYHA class III–IV 83.3% vs. 50.5%, *p* < 0.001). [63]

However, the psycho-social context, cannot be analyzed without assessing the urban context.

There is little evidence in the literature on HF management in rural settings.

In a recent study of 580 patients (58.8% male vs 41.2% female) from rural areas of the United States, depressive symptoms [*β* = 0.28, 95% confidence interval (CI): 0.16–0.45], lower perceived control (*β* = − 0.15, 95% CI: − 0.32 to − 0.08), better symptom status (*β* = − 0.11, 95% CI: − 0.13 to − 0.003), and an annual income < 20,000 $ (*β* = 0.11, 95% CI: 0.38–2.97) were negatively associated with self-care in rural residents with HF.

The authors hypothesize that depressive symptoms such as social isolation, loss of hope, and memory problems may impact treatment adherence. In addition, the costs of adequate nutrition versus high-sodium food and lack of adequate follow-up may account for the worse trend. [64]

## Conclusion and future perspectives

Table 1 represents a summary of the differences in the male/female population in sex/gender in HF.

**Table 1:** Sex/gender differences in in HF

	Female	Male
Age of onset	Later	Earlier
Specific etiology	Peripartum cardiomyopathy Chemotherapy-induced cardiomyopathy Tako-Tsubo cardiomyopathy	Uncommon
Incidence rate of HF age < 75	lower	Higher
Incidence rate of HF age > 75	Similar or higher	Similar
Pattern of HF	HFpEF	HFrEF
Comorbidities	Higher	Lower
Symptoms	Greater exercise limitation Worse quality of life Dyspnea Declining edema	Lower
Need for drug titration to reach peak benefit	Reduced	Directly proportional
Counseling for ICD	Lower	Higher
Counseling for CRT	Lower	Higher

There are important differences in epidemiology, pathophysiology, HF patterns, prognosis, and treatment.

**Fig. 1:**
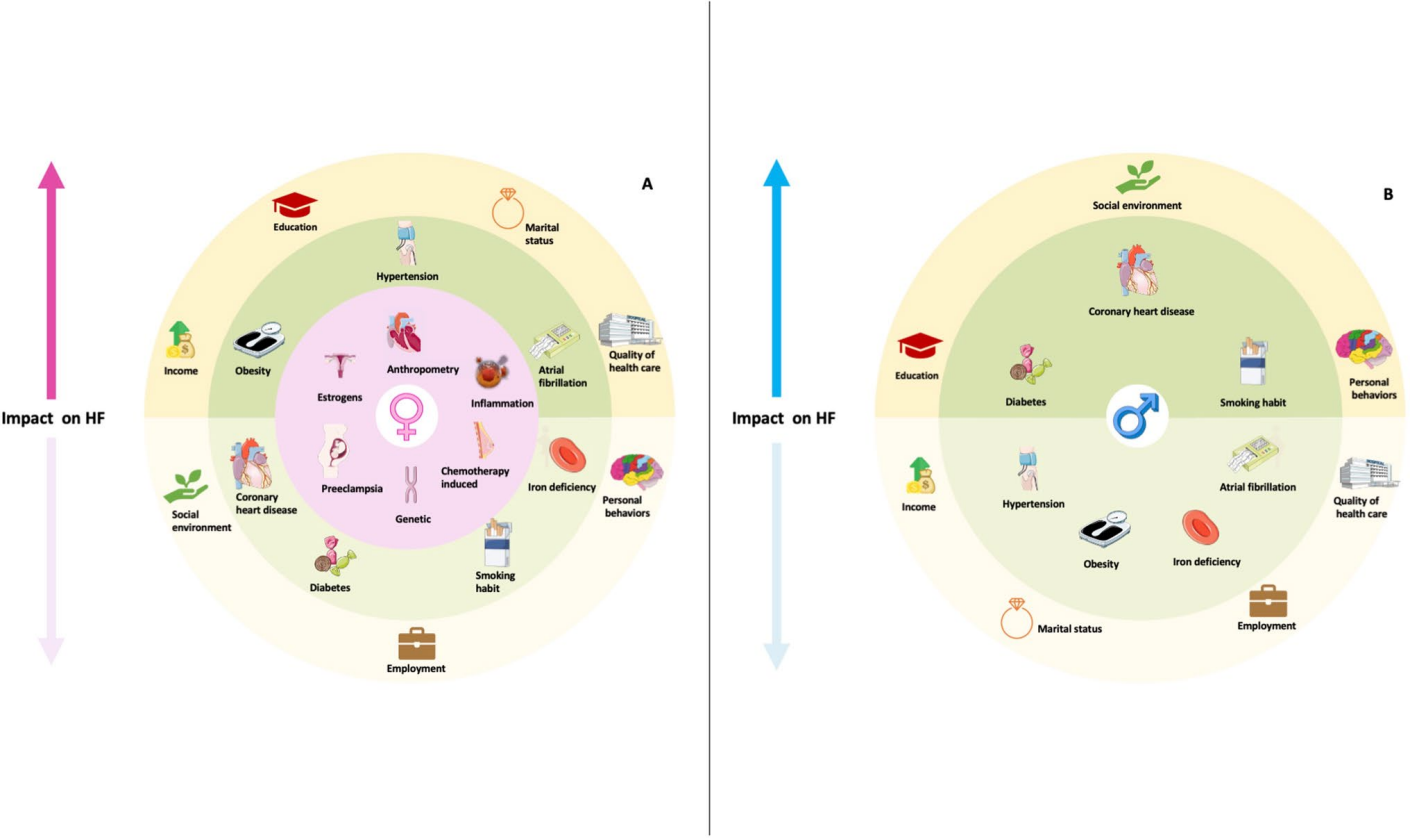
The panels depict sex specific risk factors for women (panel **A**) and men (panel **B**). Regarding women, hypertension, obesity, and atrial fibrillation play a pivotal role. For this category a moderately high risk is also associated with hormonal status, socio-cultural and behavioral issues (gender-related variables) such as marital status, income, quality of healthcare education. Men’s outcome, on the other hand, is primarily affected by a more common ischemic etiology, higher prevalence of diabetes, and bad smoking habits. In addition, more attention should be paid to social environment, personal behaviors and education

Women have a higher incidence of HFpEF due to sex-specific factors (such as anthropometry, role of estrogens, pregnancy-related cardiomyopathies), increased incidence of comorbidities, and gender-specific conditions (Fig. 1a). Men instead present a predisposition to the development of HFrEF due to a higher incidence of coronary artery disease and myocardial infarction (Fig. 1b). However, there are still gaps in the management of women with HF. The poor inclusion of women in clinical trials may have contributed to a lesser understanding of disease behavior than in men.

In fact, until 1993, the National Institutes of Health (NIH) did not consider the inclusion of women in research studies as a mandatory criterion. This condition lasted until 2015 when the NIH issued guidance requesting the scientific community to consider "sex as a biological variable" [65].

In addition, a full understanding of gender-specific factors that are studied in small populations is lacking in the literature, and only in recent years, studies have increased their focus on this issue.

Furthermore, women with low incomes seem to be exposed to higher hospital mortality and hospital readmissions, and a lower offer of substantial life-saving therapies.

Men, especially in rural settings, seem to be more affected by depression and symptoms such as social isolation that would influence their prognosis and adherence to treatment. For this reason, the existence of sex-specific risk factors (as depicted in the two panels of the figure) should be integrated by gender-related risk factors.

Understanding how society, family, and environment affect the prognosis of HF patients may help clinicians provide more appropriate levels of care. New gender-focused studies will help to tailor HF treatment and understand HF as a multifaceted disease.
